# Rapsyn facilitates recovery from desensitization in fetal and adult acetylcholine receptors expressed in a muscle cell line

**DOI:** 10.1113/JP277819

**Published:** 2019-06-17

**Authors:** Hakan Cetin, Wei Liu, Jonathan Cheung, Judith Cossins, An Vanhaesebrouck, Susan Maxwell, Angela Vincent, David Beeson, Richard Webster

**Affiliations:** ^1^ Nuffield Department of Clinical Neurosciences University of Oxford John Radcliffe Hospital Oxford UK; ^2^ Department of Neurology Medical University of Vienna Vienna Austria

**Keywords:** Neuromuscular junction, acetylcholine receptor, rapsyn, clustering

## Abstract

**Key points:**

- The physiological significance of the developmental switch from fetal to adult acetylcholine receptors in muscle (AChRs) and the functional impact of AChR clustering by rapsyn are not well studied.
- Using patch clamp experiments, we show that recovery from desensitization is faster in the adult AChR isoform.
- Recovery from desensitization is determined by the AChR isoform‐specific cytoplasmic M3–M4 domain.
- The co‐expression of rapsyn in muscle cells induced AChR clustering and facilitated recovery from desensitization in both fetal and adult AChRs. In fetal AChRs, facilitation of recovery kinetics by rapsyn was independent of AChR clustering.
- These effects could be crucial adaptations to motor neuron firing rates, which, in rodents, have been shown to increase around the time of birth when AChRs cluster at the developing neuromuscular junctions.

**Abstract:**

The neuromuscular junction (NMJ) is the site of a number of autoimmune and genetic disorders, many involving the muscle‐type nicotinic acetylcholine receptor (AChR), although there are aspects of normal NMJ development and function that need to be better understood. In particular, there are still questions regarding the implications of the developmental switch from fetal to adult AChRs, as well as how their functions might be modified by rapsyn that clusters the AChRs. Desensitization of human muscle AChRs was investigated using the patch clamp technique to measure whole‐cell currents in muscle‐type (TE671/CN21) and non‐muscle (HEK293) cell lines expressing either fetal or adult AChRs. Desensitization time constants were similar with both AChR isoforms but recovery time constants were shorter in cells expressing adult compared to fetal AChRs (*P* < 0.0001). Chimeric experiments showed that recovery from desensitization was determined by the M3–M4 cytoplasmic loops of the γ‐ and ε‐subunits. Expression of rapsyn in TE671/CN21 cells induced AChR aggregation and also, surprisingly, shortened recovery time constants in both fetal and adult AChRs. However, this was not dependent on clustering because rapsyn also facilitated recovery from desensitization in HEK293 cells expressing a δ‐R375H AChR mutant that did not form clusters in C2C12 myotubes. Thus, rapsyn interactions with AChRs lead not only to clustering, but also to a clustering independent faster recovery from desensitization. Both effects of rapsyn could be a necessary adjustment to the motor neuron firing rates that increase around the time of birth.

## Key points

- The physiological significance of the developmental switch from fetal to adult acetylcholine receptors in muscle (AChRs) and the functional impact of AChR clustering by rapsyn are not well studied.
- Using patch clamp experiments, we show that recovery from desensitization is faster in the adult AChR isoform.
- Recovery from desensitization is determined by the AChR isoform‐specific cytoplasmic M3–M4 domain.
- The co‐expression of rapsyn in muscle cells induced AChR clustering and facilitated recovery from desensitization in both fetal and adult AChRs. In fetal AChRs, facilitation of recovery kinetics by rapsyn was independent of AChR clustering.
- These effects could be crucial adaptations to motor neuron firing rates, which, in rodents, have been shown to increase around the time of birth when AChRs cluster at the developing neuromuscular junctions.

## Introduction

The muscle‐type nicotinic acetylcholine receptor in muscle (AChR) forms a heteropentameric ion channel and is composed of two α‐, one β‐, one δ‐ and one γ‐subunit in fetal muscle, which is replaced by one ε‐subunit in adult muscle. Fetal AChRs are aggregated into pre‐clusters along the muscle fibre before innervation but, on innervation, become clustered at the postsynaptic membrane (Braithwaite & Harris, 1979), which is dependent on the agrin/LRP4/MuSK/DOK7/rapsyn pathway (Tintignac *et al*. 2015). At the same time, γ‐subunit mRNA is repressed and replaced by ε‐subunit mRNA transcribed from subsynaptic nuclei (Brenner *et al*. 1990; Croxen *et al*. 2001). In human muscle, the switch from fetal to adult AChRs is largely completed in the third trimester of gestation (Hesselmans *et al*. 1993), although fetal AChRs continue to be expressed at low levels in adult muscle (MacLennan *et al*. 1997). By contrast, in mice, the switch from fetal to adult AChR mRNA expression takes place postnatally and appears to be complete within 2 weeks with no easily detectable γ‐subunit expression in adult muscle (Sakmann & Brenner, 1978).

Fetal and adult AChRs differ in various functional aspects. It is already known that the fetal isoform has a longer mean open time and a lower conductance compared to the adult receptor (Mishina *et al*. 1986) and that agonist affinities are higher in fetal AChRs (Nayak *et al*. 2014). It has also been suggested that the higher affinity of the fetal AChR for choline, together with the longer mean open time, results in substantially larger responses of fetal AChRs in prenatal stages, when choline is present at higher serum concentration levels than in adults (Nayak *et al*. 2014). Previous data on desensitization and recovery from desensitization show that both depend on phosphorylation of the M3–M4 cytoplasmic loop of various AChR subunits (Huganir *et al*. 1986; Middleton *et al*. 1986; Hopfield *et al*. 1988; Paradiso & Brehm, 1998), although there is varying evidence for a difference in desensitization and recovery kinetics between the two isoforms (Auerbach & Akk, 1998; Paradiso & Brehm, 1998; Jahn *et al*. 2001; Krampfl *et al*. 2002).

The interaction of AChRs with other proteins and between AChR pentamers in the process of clustering could also influence desensitization and recovery kinetics. Rapsyn is a 43 kDa membrane‐associated cytoplasmic protein colocalized with AChRs at the neuromuscular junction (NMJ) and shown to interact with the M3–M4 cytoplasmic loops of all AChR subunits (Maimone & Merlie, 1993; Huebsch & Maimone, 2003) via up to three bridges, which is the minimum number required to form a 2‐D network (Zuber & Unwin, 2013). A coiled‐coil domain encompassing the rapsyn amino acid sequence 298–331 was shown to be crucial for AChR clustering (Ramarao & Cohen, 1998).

To explore the potential role of rapsyn or receptor clustering on AChR function, we used the whole‐cell, patch clamp technique and studied desensitization and recovery from desensitization in human fetal and adult AChRs expressed in both muscle and non‐muscle cell lines. Chimeric AChRs with exchanged M3–M4 cytoplasmic loops between the γ‐ and ε‐subunit were constructed to address the role of the cytoplasmic domain on desensitization and recovery kinetics, and rapsyn was co‐expressed in the muscle cell lines to determine to what extent AChR clustering modifies fetal and adult AChR functions.

## Methods

### Cell cultures

TE671 is a rhabdomyosarcoma cell line with muscle‐like properties (Stratton *et al*. 1989). CN21 cells derive from the TE671 cell line and have been stably transfected with the cDNA of the human AChR ε‐subunit, such that the adult AChR isoform is expressed in high numbers with only a remaining small fraction of fetal AChRs (Beeson *et al*. 1996). TE671 and CN21 were cultured at 37°C and 5% CO_2_ in Dulbecco‐modified essential medium (DMEM) (Sigma‐Aldrich, St Louis, MO, USA) supplemented with 10% fetal calf serum (FCS) and 1% antibiotics/anti‐mycotics [i.e. penicillin G, streptomycin and amphotericin B (PSA)] (Invitrogen, Carlsbad, CA, USA). HEK293 cells were maintained at 37°C and 5% CO_2_ in DMEM supplemented with 10% FCS and 1% PSA. C2C12 were maintained at 37°C and 5% CO_2_ in DMEM supplemented with 15% fetal calf serum and 1% antibiotics/anti‐mycotics and differentiated to form myotubes for 4–7 days in DMEM supplemented with 2% fetal calf serum and 1% antibiotics/anti‐mycotics.

### Plasmids and constructs

Complementary DNAs encoding human AChR α‐ (P3A negative isoform), β‐, δ‐, γ‐ and ε‐subunits were cloned into pcDNA3.1‐hygro. Complementary DNA encoding human rapsyn (transcript: ENST00000298854.6) was cloned into pIRES2‐EGFP enabling the simultaneous expression of rapsyn and EGFP (pIRES2‐RAPSN‐EGFP). Chimeric subunits were engineered using the QuickChange Multi Site‐Directed Mutagenesis Kit (Agilent Technologies Inc., Santa Clara, CA, USA). Restriction enzymes were purchased from New England Biolabs (Beverly, MA, USA). A *BsrG*I restriction site was cloned into the 5′‐end of γ‐ and ε‐M3–M4 cytoplasmic loops using the primers 5′‐TGTGGTTGTGCTCAATGT ACACTTGCGGTCTCCACAC‐3′ and 5′‐GCGTCATCGT GCTCAATGTACACCAGCGGACGCCCAC‐3′, respectively, and an *Age*I restriction site was cloned into the 3′‐end of γ‐ and ε‐M3–M4 cytoplasmic loops using the primers 5′‐GGGCCGAGTGCTGGACCGGTTCTGCTTCCTGGC CATG‐3′ and 5′‐TGGGGAATGCCCTTGACCGGTTCTG CTTCTGGGCCGC‐3′, respectively. Then, using *BsrG*I and *Age*I, the 145 amino acids long γ‐M3–M4 cytoplasmic loop was cloned into the ε‐subunit and the 128 amino acids long ε‐M3–M4 cytoplasmic loop was cloned into the γ‐subunit. *BsrG*I and *Age*I restriction sites were mutated back using primers 5′‐CTGTGGTTGTGCTCAATGTCT CCCAGCGGACGCCCAC‐3′ and 5′‐GGGAATGCCCTT GACAACGTCTGCTTCCTGGCCATGC‐3′ for the chimeric γ‐subunit, and primers 5′‐TGCGTCATCGTGCTC AACGTGTCCTTGCGGTCTCCAC‐3′ and 5′‐GGCCGA GTGCTGGACCGCATCTGCTTCTGGGCCGCTC‐3′ for the chimeric ε‐subunit. Complementary DNA encoding the human δ‐subunit (transcript: ENST00000298854.6) was cloned into pBABE‐puro and δ‐R375H (numbering according to the mature polypeptide without the signal sequence) was generated by site‐directed mutagenesis using QuickChange (Stratagene, La Jolla, CA, USA). Complementary DNAs were Sanger sequenced following mutagenesis to confirm the presence of exchanged M3–M4 cytoplasmic loops or of the mutated residue and the absence of additional variants.

### Transfections

To express AChR isoforms in HEK293, cells were seeded at 10^6^ cells per well in six‐well plates. At 60–80% confluence the next day, cells were exposed to a mix of 3 µg of cDNA, 20% glucose and PEI in 2 ml of growth medium. Human muscle AChR α‐, β‐, δ‐ and either γ‐subunit cDNA were used for fetal AChR expression (HEK293‐γ) or ε‐subunit cDNA for adult AChR expression (HEK293‐ε). Cells were co‐transfected with pEGFP‐N1 (Invitrogen, Carlsbad, CA, USA), which contains cDNA encoding EGFP, or with pEGFP‐RAPSN for the co‐expression of EGFP‐tagged rapsyn. After 5–7 h of exposure, HEK293 were detached from the plates using trypsin/EDTA, centrifuged at 1200 *g* for 4 min, resuspended in growth medium and plated onto poly‐l‐lysine‐coated glass coverslips.

To express rapsyn in TE671/CN21, cells were seeded at 2 × 10^5^ cells per well in six‐well plates. The next day, cells were detached from the plates using trypsin/EDTA, centrifuged at 1200 *g* for 4 min and resuspended in pre‐warmed growth medium without PSA. A corresponding amount of the cell suspension containing 1.25 × 10^6^ cells was again centrifuged at 1200 *g* for 5 min and resuspended in 250 µL of resuspension buffer R containing 20 µg of DNA at a concentration of 1 µg µL^–1^. Cells were electroporated with either pIRES2‐EGFP (‘no rapsyn’) or pIRES2‐RAPSN‐EGFP (‘with rapsyn’) at 1100 V and a pulse width of 30 ms using the NEON transfection system (MPK 5000; Invitrogen). To increase AChR expression levels, another group of TE671 and CN21 was additionally transfected with cDNA encoding fetal and adult AChR subunits, respectively. Immediately after electroporation, cells were resuspended in pre‐warmed growth medium without PSA and plated onto poly‐l‐lysine‐coated glass coverslips. The medium was replaced by growth medium with PSA the next day.

### Generation of CHRND‐knockout (KO) C2C12 cells

CHRND‐KO cells were generated using the CRISPR‐Cas9n system (Ran *et al*. 2013). To introduce loss‐of‐function mutations in the CHRND of C2C12 cells (i.e. a mouse muscle cell line) (Yaffe & Saxel, 1977), 10 µg of the CRISPR plasmid containing the guides A and B was electroporated into C2C12 myoblasts without the repair template to promote spontaneous in‐dels that would cause a frameshift in the δ‐subunit transcript. Myoblasts of 12 clones were differentiated into myotubes for agrin‐induced AChR clustering to preliminarily identify clones that do not form AChR clusters. The clone KO6 was identified to differentiate into myotubes without forming AChR clusters after incubation with a 1:100 dilution of medium harbouring full‐length human agrin and was thus chosen for further experiments. Sequencing of the genomic PCR product of KO6 (a single band at ∼750 bp on agarose electrophoresis) identified three variants for *CHRND*: two different 13 bp deletions c.del1136_1148TGTTTGAGAAGCA (c.del1136_1148) and c.del1131_1143CCTCATGTTTGAG (c.del1131_1143) and a 25 bp deletion c.del1132_1156CTCATGTTTGA GAAGCAATCAGAGC (c.del1132_1156). The three different variants of CHRND identified in the cell line KO6 most probably derived from the polyploidy nucleus frequently observed in C2C12 (Burattini *et al*. 2004). All three variants cause a frameshift and lead to 31, 28 and 33 missense amino acids, respectively, followed by a premature stop codon. The variants lead to the loss of the entire M4 transmembrane domain and result in a non‐functional δ‐subunit.

### AChR clustering assay

C2C12 were differentiated to myotubes by growth factor depletion. C2C12 myotubes were incubated for 16 h with full‐length human agrin diluted 1:100 in differentiation medium. AChR clusters were stained with Alexa Fluor® 594 conjugated α‐bungarotoxin (catalogue no. B13423; Invitrogen) diluted in 1:1000 in differentiation medium to a final concentration of 1 µg mL^–1^ for 60 min at 37°C and 5.5% CO_2_. They were then washed 3 × 5 min with differentiation medium and fixed for 20 min with 3% paraformaldehyde at room temperature in the dark. Myotubes were washed with PBS and stored in PBS at 4°C.

### Quantification of AChR clusters formed on C2C12 myotubes

Images used for the quantification of AChR clusters labelled with Alexa Fluor® 594 conjugated α‐bungarotoxin (Invitrogen) on cultured C2C12 myotubes were acquired at 20× magnification using an IX71 fluorescence microscope (Olympus, Tokyo, Japan) with SimplePCI software (Digital Pixel, Brighton, UK). The number, length, average area and total area of AChR clusters larger than 2.5µm^2^ per field were quantified by Fiji macro analysis (NIH, Bethesda, MD, USA).

### Fluorescence and confocal microscopy

Microscopy was performed on a LSM 880 confocal microscope (Carl Zeiss, Oberkochen, Germany). Between 2 or 3 days after transfection, cells were incubated with mAb C7 (Jacobson *et al*. 1999) for 1 h at room temperature, which binds the extracellular domain of the AChR δ‐subunit, diluted 1:1000 in staining medium (DMEM containing 20 mm Hepes and 1% BSA), before being washed three times with staining medium. Cells were fixed with 3% paraformaldehyde at room temperature for 10 min, washed three times with PBS and incubated with secondary antibody Alexa Fluor® 594 goat anti‐mouse IgG (H+L) (catalogue no. A11005; Invitrogen; RRID:AB_141372) diluted 1:750 in staining medium. Cells were washed three times in PBS and mounted in fluorescence mounting medium (Dako Cytomation, Glostrup, Denmark). Images were captured using the ZEN System imaging software (Carl Zeiss).

### Cell‐surface ^125^I‐α‐bungarotoxin binding assay

The levels of AChRs on the surfaces of cells cultured in 6‐well plates were measured by the ^125^I‐α‐bungarotoxin (^125^I‐α‐BuTx) binding assay. For transfected HEK293 cells, the assay was performed on cells 48 h after transfection. To ensure the reproducibility of results, each assay analysed duplicate wells of cells for each condition. In the assay, each well was washed three times with PBS, followed by incubations with 500 µL of ^125^I‐α‐BuTx at 10^6^ cpm mL^–1^ diluted in blocking solution (DMEM containing 20 mm Hepes and 1% bovine serum albumin, BSA) for 1 h at room temperature with gentle rocking. Subsequently, the cells were washed 3 × 5 min each with 1 mL of PBS and then dissolved in 500 µL of protein extraction buffer. The cell extracts were transferred to an Eppendorf tube and the amounts of ^125^I‐α‐BuTx bound were quantified by a gamma‐counter. Untransfected HEK293 cells were used for the estimation of the background level of radioactivity, which was subtracted from the cell‐surface ^125^I‐α‐BuTx binding in transfected cells to calculate AChR expression levels.

### Electrophysiology

Both TE671/CN21 and HEK293 were patched between 2 or 3 days after transfection. All experiments were performed at room temperature using the whole‐cell, patch clamp configuration. Recording pipettes were made of thin‐walled borosilicate glass (GC150TF‐10; Harvard Apparatus, Cambridge, MA, USA). Pipette tips were fire‐polished to a final resistance of ∼3 MΩ (MF‐900 Microforge; Narishige, London, UK). Extracellular solutions contained (in mm): 150 NaCl, 2.8 KCl, 10 Hepes, 2 MgCl_2_, 2 CaCl_2_ and 10 glucose with pH adjusted to 7.4 using NaOH. Pipette solutions contained (in mm): 4 NaCl, 144 KCl, 10 Hepes, 2 MgCl_2_, 2 ATP and 10 EGTA with pH adjusted to 7.2 using KOH. All measurements were performed at a holding potential of −60 mV. Currents were amplified using an Axopatch‐1D amplifier (Molecular Devices, Sunnyvale, CA, USA) and, after filtering at 5 kHz, sampled to hard disk at 25 kHz. Series resistance was either corrected using the amplifier‐based compensation circuit during the recording or a posteriori by a software‐based computational algorithm as implemented in ChannelLab (Synaptosoft Inc., Fort Lee, NJ, USA) (Traynelis, 1998). Residual holding potential errors were <2 mV.

Fast solution exchange was accomplished using the modified HSSE‐2/3 application system (ALA Scientific Instruments, Farmingdale, NY, USA). A two‐barrel perfusion pipette with a tip diameter of ∼300 µm was used to switch between control and test solutions, both of which consisted of an agonist containing solution for receptor stimulation (extracellular solution with 1 mm ACh) and an agonist‐free solution for agonist removal (extracellular solution). Application times were fast with 10–90% rise times <1 ms in open pipette experiments and 10–90% current rise times <10 ms in the whole‐cell configuration.

### Analysis of desensitization and recovery from desensitization

**Figure 1:**
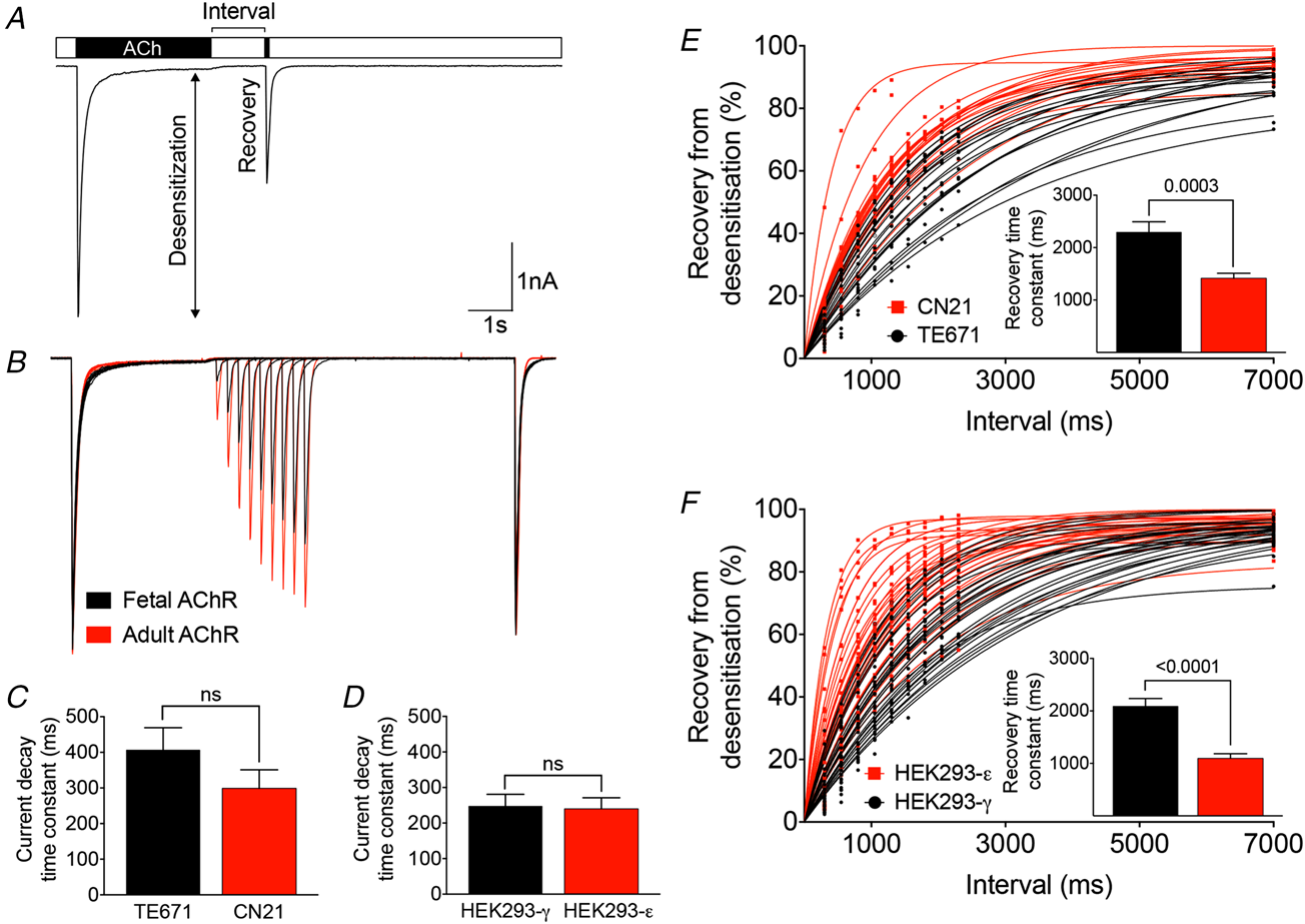
Desensitization and recovery kinetics in fetal and adult AChRs *A*, receptor stimulation protocol including ten sweeps of 25 s in duration, each of which consisted of two consecutive ACh pulses of 1 mm concentration. A desensitizing pulse of 3 s in duration was followed by a 50 ms test pulse after an increasing interval with each sweep. *B*, averaged recordings from three HEK293 cells expressing fetal AChRs (black trace) and three HEK293 cells expressing adult AChRs (red trace). For better comparison, traces were normalized to the peak current amplitude of the desensitizing pulse in the corresponding first sweep. Ten sweeps are overlaid displaying similar current decay between fetal and adult AChRs and faster recovery from desensitization in adult AChRs. *C*, in the muscle‐type cell line, mean weighted current decay time constants were 407 ± 62 ms in TE671 (*n* = 20) and 299 ± 52 ms in CN21 (*n* = 20). *D*, in the non‐muscle cell line, mean weighted current decay time constants were 248 ± 33 ms in HEK293‐γ (*n* = 30) and 240 ± 31 ms in HEK293‐ε (*n* = 30). *E*, in the muscle‐type cell line, mean recovery time constants were 2297 ± 197 ms in TE671 (*n* = 20) and 1417 ± 93 ms in CN21 (*n* = 20). In two CN21 cells, recovery from desensitization was markedly fast (*E*). Neither the presence, nor the absence of these two cells made a difference to the statistical significance (mean recovery time constant of 1500 ± 75 ms in CN21 (*n* = 18), *P* = 0.0009 after exclusion of outliers). *F*, in the non‐muscle cell line, mean recovery time constants were 2093 ± 142 ms in HEK293‐γ (*n* = 30) and 1097 ± 88 ms in HEK293‐ε (*n* = 30). Data represent the mean ± SEM. [Color figure can be viewed at wileyonlinelibrary.com]

Receptor stimulation protocols included ten sweeps of 25 s in duration, each of which consisted of two consecutive ACh pulses. A ‘desensitizing’ pulse of 3 s in duration was followed by a 50 ms ‘test’ pulse. The interval between pulses increased with each sweep from 300 ms to 7 s until full recovery from desensitization (Fig. 1 *A* and *B*). Desensitization was analysed by fitting one or two exponential components to the current decay phase during the application of the desensitizing pulse. For better comparison of desensitization between groups, weighted current decay time constants were calculated according to: (τ_1_ × *A*_1_ + τ_2_ × *A*_2_)/(*A*_1_ × *A*_2_), where τ_x_ is the decay time constant for a particular component of the curve and *A*_x_ is the amplitude of the corresponding component. Recovery from desensitization was analysed as the ratio between peak current amplitudes of the test and desensitizing pulse in ten sweeps plotted against the interval between desensitizing and test pulse. Recovery time constants were determined by fitting one or two exponential components to the data points. Clampfit, version 10.5 (Molecular Devices) or Prism, version 7.0a (GraphPad Software Inc., San Diego, CA, USA) were used for exponential curve fitting and current decay or recovery time constant calculation, respectively.

### Statistical analysis

Detailed experimental designs of both immunofluorescence and electrophysiological data are provided in the Results, including the number of cells used in the experiments. All data were analysed using Prism, version 7.0a (GraphPad Software Inc.). The results are reported as the mean ± SEM and compared using a two‐tailed unpaired *t* test. The degrees of freedom are stated for each test.

## Results

### Desensitization is not different between fetal and adult AChRs but recovery from desensitization is faster in adult AChRs

First, we used the TE671 and CN21 muscle‐type cell lines; the TE671 cells are derived from a rhabdomyosarcoma and only express the fetal AChR isoform (Stratton *et al*. 1989). The CN21 cells were derived from the TE671 cells by permanent introduction of the gene for the ε‐subunit so that they predominantly express adult AChRs (Beeson *et al*. 1996). We then compared the results with those in the human non‐muscle embryonic kidney cells (HEK293), in which we expressed the two AChR isoforms independently. Whole‐cell currents were recorded from both muscle‐type and non‐muscle cells expressing fetal and adult AChRs on their surface (Fig. 1 *A*). Desensitization was assessed by the calculation of current decay time constants during a 3 s pulse of 1 mm ACh, a period sufficient to enable almost complete desensitization [desensitized current fraction of 93.0 ± 1.3% in TE671 (*n* = 20), 93.1 ± 1.8% in CN21 (*n* = 20), 94.4 ± 1.2% in HEK293‐γ (*n* = 30) and 93.0 ± 1.4% in HEK293‐ε (*n* = 30)]. Current decay was fitted best by the sum of two exponentials and weighted time constants were not significantly different between fetal and adult AChRs in either cell line (TE671/CN21: *t*_38_ = 1.34, *P* = 0.1899; HEK293: *t*_58_ = 0.18, *P* = 0.8604) (Fig. 1 *C* and *D*).

Recovery from desensitization was measured as the ratio between peak current amplitudes of the test and desensitizing pulse in ten sweeps with increasing intervals ranging from 300 ms to 7 s, a period after which the majority of receptors had recovered from desensitization [87.7 ± 1.2% in TE671 (*n* = 20), 93.4 ± 0.7% in CN21 (*n* = 20), 91.6 ± 0.8 in HEK293‐γ (*n* = 30) and 93.1 ± 0.7 in HEK293‐ε (*n* = 30)]. Recovery ratio plots could be fitted best by a single exponential that resulted in shorter recovery time constants in adult compared to fetal AChRs in both the muscle‐type (*t*_38_ = 4.04, *P* = 0.0003) and non‐muscle cell lines (*t*_58_ = 5.96, *P* < 0.0001) (Fig. 1 *B*, *E* and *F*). In two CN21 cells, recovery from desensitization was markedly fast (Fig. 1 *E*). After exclusion of these outliers, the mean recovery time constant remained faster in adult compared to fetal AChRs (*t*_36_ = 3.63, *P* = 0.0009).

### Recovery from desensitization is determined by the AChR isoform‐specific M3–M4 cytoplasmic loop

**Figure 2:**
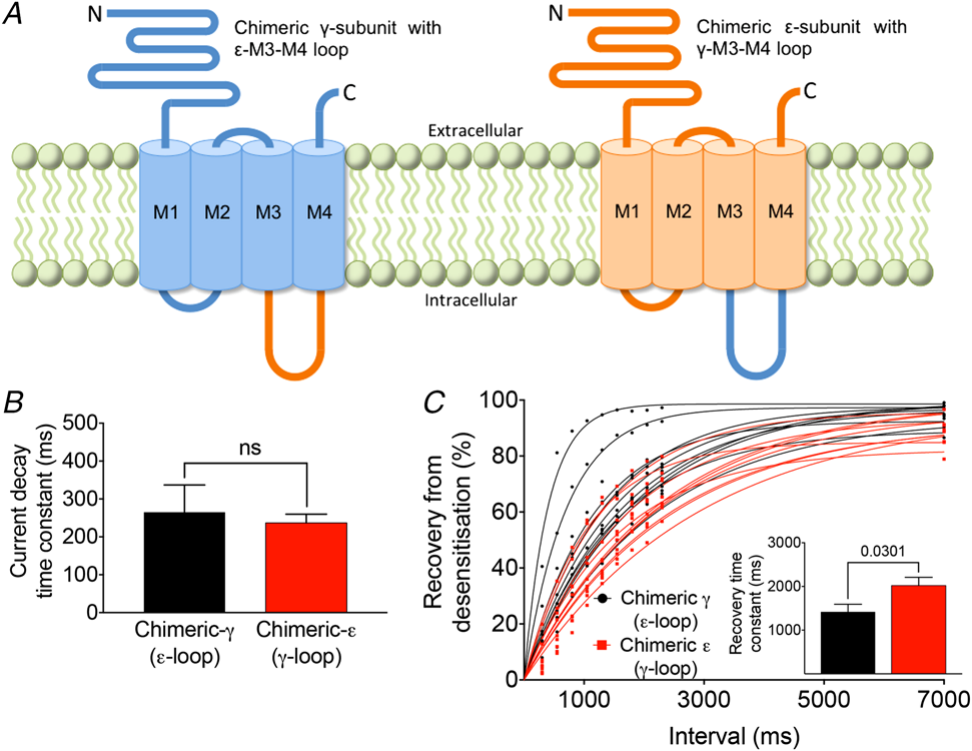
Chimeric AChRs with exchanged M3–M4 cytoplasmic loops between γ‐ and ε‐subunits *A*, chimeric γ‐subunits (blue) with ε‐M3–M4 cytoplasmic loop (orange) and chimeric ε‐subunits (orange) with γ‐M3–M4 cytoplasmic loop (blue). Both M3–M4 loops differ at 94/146 positions. *A*, mean weighted current decay time constants were 265 ± 72 ms in chimeric fetal (*n* = 10) and 237 ± 23 ms in chimeric adult (*n* = 10) AChRs. *C*, mean recovery time constants were 1419 ± 174 ms in chimeric fetal (*n* = 10) and 2022 ± 188 ms in chimeric adult (*n* = 10) AChRs. In two cells expressing chimeric fetal AChRs, recovery from desensitization was markedly faster. After exclusion of these two cells, the mean recovery time constant remained faster in chimeric fetal AChRs (1634 ± 121 ms, *n* = 8), although the difference compared to chimeric adult AChRs was no longer significant (*t*_16_ = 1.64, *P* = 0.1215). There was no obvious explanation for the markedly faster recovery from desensitization in the two cells in (*C*). Data represent the mean ± SEM. [Color figure can be viewed at wileyonlinelibrary.com]

The cytoplasmic loops between the M3 and M4 transmembrane domains of γ‐ and ε‐subunits differ by 94/146 amino acids. We constructed chimeric AChRs with exchanged M3–M4 loops between γ‐ and ε‐subunits (Fig. 2 *A*) and expressed them in HEK293 cells. Current decay time constants were not different between chimeric receptors (*t*_18_ = 0.37, *P* = 0.7154) (Fig. 2 *B*), although recovery from desensitization was faster in chimeric fetal AChRs/ε‐M3–M4 loop than in chimeric adult AChRs/γ‐M3–M4 loop (*t*_18_ = 2.35, *P* = 0.0301) (Fig. 2 *C*). In chimeric fetal AChRs with the ε‐M3–M4 loop, there were two outliers with markedly faster recovery time constants. After exclusion of these two cells, the mean recovery time constant remained faster in chimeric fetal AChRs but the difference to chimeric adult AChRs was no longer significant (*t*_16_ = 1.64, *P* = 0.1215). However, because there was no obvious explanation for the markedly faster recovery from desensitization in the two cells, they were not excluded from the analysis. Thus, the ε‐subunit M3–M4 loop transferred fast recovery to the fetal AChR and the γ‐subunit M3–M4 loop transferred slow recovery to the adult AChR.

### Rapsyn induces AChR aggregation and facilitates recovery from desensitization of endogenous fetal and adult AChRs

**Figure 3:**
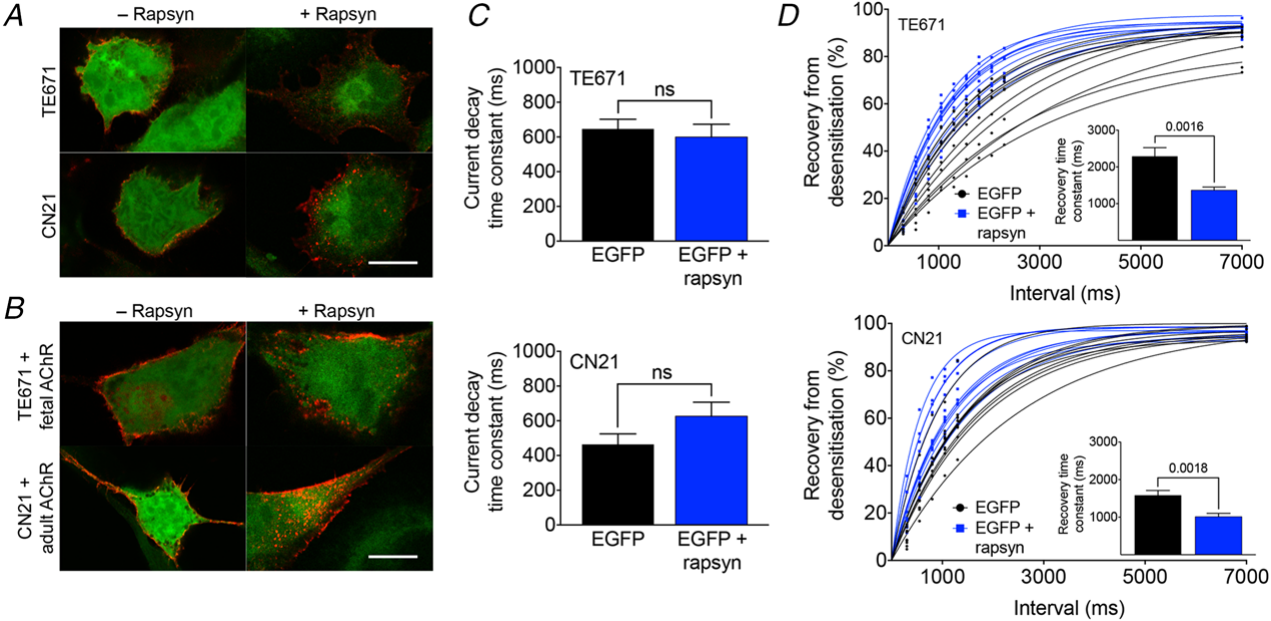
Effect of rapsyn on desensitization and recovery kinetics in fetal and adult AChRs *A*, in TE671/CN21 cells, the co‐transfection of rapsyn cDNA was associated with AChR aggregation on the cell surface. Scale bar = 10 µm. *B*, the number and size of AChR aggregates increased after additional transfection of TE671 and CN21 with the corresponding AChR isoform subunits. Scale bar = 10 µm. *C*, rapsyn had no effect on desensitization in TE671 and CN21. Mean weighted current decay time constants were 646 ± 56 ms without rapsyn (*n* = 10) and 600 ± 73 ms with rapsyn (*n* = 10) in TE671, and 464 ± 61 ms without rapsyn (*n* = 10) and 627 ± 80 ms with rapsyn (*n* = 10) in CN21. *D*, recovery from desensitization was faster in both TE671 and CN21 with rapsyn. Mean recovery time constants were 2289 ± 234 ms without rapsyn (*n* = 10) and 1368 ± 85 ms with rapsyn (*n* = 10) in TE671, and 1584 ± 128 ms without rapsyn (*n* = 10) and 1014 ± 89 ms with rapsyn (*n* = 10) in CN21. Data represent the mean ± SEM. [Color figure can be viewed at wileyonlinelibrary.com]

Immunofluorescence staining of TE671/CN21 muscle‐type cell lines confirmed rapsyn‐induced AChR aggregation (Fig. 3 *A*), although the level of AChR aggregation was low, probably as a result of lower AChR expression levels in TE671/CN21 cells compared to transfected HEK293 cells. After increasing AChR expression levels in TE671/CN21 by additional transfection with cDNA of all of the corresponding AChR isoform subunits (TE671 + fetal AChRs and CN21 + adult AChRs, respectively), the level of AChR aggregation also increased and helped to distinguish cells with and without rapsyn visually (Fig. 3 *B*). As a result of the heterogeneity and small size of the AChR aggregates ranging between 0.2 and 0.5 µm, their quantification by Fiji macro analysis was not feasible.

In whole‐cell patch clamp experiments, current decay was not significantly different after transfection with human rapsyn cDNA in either TE671 cells (*t*_18_ = 0.50, *P* = 0.6232) or CN21 cells (*t*_18_ = 1.62, *P* = 0.1226) (Fig. 3 *C*). Recovery from desensitization, by contrast, was significantly faster in TE671 (*t*_18_ = 3.70, *P* = 0.0016) and CN21 cells (*t*_18_ = 3.66, *P* = 0.0018) co‐transfected with rapsyn cDNA (Fig. 3 *D*). In both groups, recovery ratio plots could be fitted best by a single exponential. Thus, rapsyn facilitates not only receptor aggregation but also recovery from desensitization in muscle‐type cells expressing AChRs.

### Rapsyn can facilitate AChR recovery kinetics in a non‐clustering AChR mutant

The facilitation of AChR recovery kinetics by rapsyn could be mediated by the interaction of rapsyn with individual subunits of the receptor or depend on AChR clustering. To attempt to distinguish these possibilities, we took advantage of a naturally occurring congenital myasthenic syndrome (CMS) mutation (δ‐R375H), located in the M3–M4 cytoplasmic loop of the δ‐subunit.

**Figure 4:**
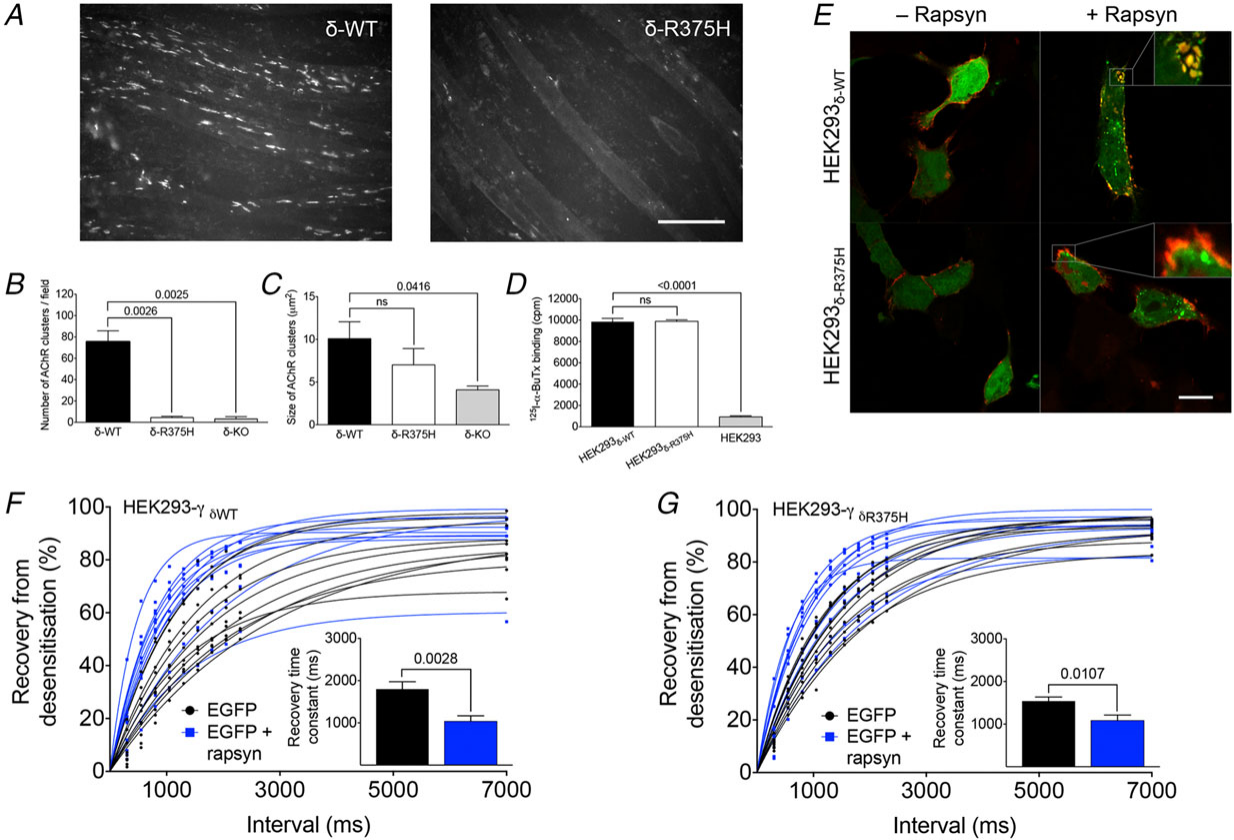
Functional impact of rapsyn on AChR function in the absence of AChR clustering *A*, comparison of C2C12 myotubes expressing wild‐type AChRs or AChRs bearing the δ‐R375H. The mutation was associated with the failure of the AChRs to cluster. Scale bar = 100 µm. *B*, mean cluster numbers were 75.3 ± 10.5 per vision field in C2C12_δ‐WT_ (*n* = 3) and 4.4 ± 1.2 per vision field in C2C12_δ‐R375H_ (*n* = 3). In C2C12_δ‐KO_, the mean cluster number per vision field was 3.2 ± 2.1 (*n* = 3). *C*, there was no statistically significant difference in the size of remaining clusters in C2C12_δ‐R375H_ (7.0 ± 1.9 µm^2^, *n* = 3) and C2C12_δ‐WT_ (10.1 ± 2.0 µm^2^, *n* = 3). *D*, surface expression of AChR_δ‐R375H_ was robust and similar to AChR_δ‐WT_ when tested in HEK293 cells [^125^I‐α‐BuTx binding of 9882 ± 143 cpm in HEK293_δ‐R375H_ (*n* = 3) and 9847 ± 312 cpm in HEK293_δ‐WT_ (*n* = 3)]. *E*, by contrast to HEK293‐γ_δ‐WT_ co‐transfected with rapsyn, where AChR aggregates were clearly visible (upper right), AChR aggregation was inconsistent in HEK293‐γ_δ‐R375H_ despite the co‐localization of rapsyn with AChR_δ‐R375H_ (lower right). Scale bar = 10 µm. Recovery from desensitization was analysed in HEK293 expressing wild‐type (*F*) or mutant AChRs (*G*). In both, the co‐transfection of rapsyn cDNA was associated with significantly faster recovery kinetics. In HEK293‐γ_δ‐WT_, mean recovery time constants were 1799 ± 176 ms without rapsyn (*n* = 10) and 1037 ± 132 ms with rapsyn (*n* = 10). In HEK293‐γ_δ‐R375H_, mean recovery time constants were 1541 ± 95 ms without rapsyn (*n* = 10) and 1085 ± 129 ms with rapsyn (*n* = 10). Data represent the mean ± SEM. [Color figure can be viewed at wileyonlinelibrary.com]

The impact of δ‐R375H on AChR clustering was analysed in C2C12 myotubes (a mouse muscle cell line expressing fetal AChRs) after knocking‐out the mouse CHRND gene that codes for the AChR δ‐subunit (C2C12_δ‐KO_) using CRISPR‐Cas9 technology and then transfecting C2C12_δ‐KO_ with either human δ‐WT (C2C12_δ‐WT_) or human δ‐R375H cDNA (C2C12_δ‐R375H_). Human agrin was applied to compare AChR clustering between C2C12_δ‐WT_ and C2C12_δ‐R375H_. The presence of the δ‐R375H was associated with markedly reduced AChR cluster numbers (*t*_4_ = 6.71, *P* = 0.0026), similar to those found in C2C12_δ‐KO_ (Fig. 4 *A*–*C*).

The impact of the δ‐R375H on AChR surface expression was tested by transfection of cDNAs encoding the corresponding human AChR subunits into HEK293 cells and counting AChR‐binding of radioactive ^125^I‐α‐BuTx. Surface expression of HEK293_δ‐R375H_ was robust and similar to HEK293_δ‐WT_ (*t*_4_ = 0.10, *P* = 0.9244) (Fig. 4 *D*). To assess the co‐localization of rapsyn with AChRs, HEK293 cells expressing human fetal AChRs (HEK293‐γ) were co‐transfected with pEGFP‐RAPSN for the expression of EGFP‐tagged rapsyn. This resulted in clearly discernible AChR aggregation in HEK293‐γ_δ‐WT_ co‐transfected with rapsyn (Fig. 4 *E*, upper right), whereas, in HEK293‐γ_δ‐R375H_, AChR distribution was less clearly aggregated after the co‐transfection with rapsyn (Fig. 4 *E*, lower right). The AChR aggregates in HEK293 cells were generally smaller and appeared as rudimentary forms of the large AChR clusters in C2C12 myotubes. However, rapsyn was still found to co‐localize with AChRs in HEK293‐γ_δ‐R375H_, even in the absence of clear AChR aggregates (Fig. 4 *E*, lower right), suggesting a direct interaction between rapsyn and the mutant AChR. It was thus concluded that the reduction of AChR cluster numbers in C2C12 was a result of the inability of mutant AChRs to produce well‐formed clusters rather than resulting from reduced surface expression of mutant AChRs or from the inability of mutant AChRs to interact with rapsyn.

We then tested recovery from desensitization in HEK293 cells expressing fetal AChRs including either the WT (HEK293‐γ_δ‐WT_) or mutant δ‐subunits (HEK293‐γ_δ‐R375H_) co‐transfected with rapsyn cDNA. Surprisingly, recovery time constants were shorter irrespective of the clustered state of the AChRs (δ‐WT: *t*_18_ = 3.46, *P* = 0.0028; δ‐R375H: *t*_18_ = 2.85, *P* = 0.0107) (Fig. 4 *F* and *G*). Thus, facilitation of AChR recovery kinetics by rapsyn does not depend on receptor clustering but, instead, on the simple interaction of the receptor with rapsyn.

## Discussion

The existence of subtle differences in structure and pharmacology of fetal and adult AChRs at mammalian NMJs was established in the 1970s (Green *et al*. 1975), although the functional implications are still not yet fully understood despite the increasing number of recognized disorders of neuromuscular transmission (Cetin & Vincent, 2018; Rodriguez Cruz *et al*. 2018). We compared desensitization and recovery kinetics between human fetal and adult AChRs expressed in muscle‐type and non‐muscle cell lines using whole‐cell patch clamp recordings and an optimized perfusion system, which enabled direct and accurate comparisons. Desensitization did not differ between the two isoforms but recovery from desensitization was faster in the adult AChR and dependent on the isoform‐specific M3–M4 cytoplasmic loop, suggesting a role for phosphorylation. Nevertheless, rapsyn co‐expression facilitated recovery kinetics of both isoforms and, unexpectedly, this effect was independent of AChR clustering.

Desensitization of the nicotinic AChR at the NMJ was described over 60 years ago and shown to occur within seconds of agonist application and to recover within seconds of agonist removal (Katz & Thesleff, 1957). More recent studies characterized desensitization in fetal and adult AChRs with time constants ranging from tens of milliseconds (Paradiso & Brehm, 1998; Jahn *et al*. 2001; Krampfl *et al*. 2002) to several seconds (Hopfield *et al*. 1988; Wagoner & Pallotta, 1988) or even minutes (Chesnut, 1983). Desensitization is a function of agonist concentration (Dilger & Liu, 1992) and these differences may have resulted from varying agonist concentrations used in different studies. Phosphorylation of the M3–M4 cytoplasmic AChR loop has also been shown to affect desensitization and recovery from desensitization (Huganir *et al*. 1986; Hopfield *et al*. 1988; Hoffman *et al*. 1994; Paradiso & Brehm, 1998). It is possible that post‐translational AChR modification influenced desensitization or different applications of the patch clamp technique could have contributed to the differences found in previous studies (Auerbach & Akk, 1998). In the present study, we attempted to avoid these possibilities by using the whole‐cell method and making direct comparisons between fetal and adult AChRs expressed in both muscle‐type (TE671/CN21) and non‐muscle (HEK293) cells, in which desensitization was found to be similar in fetal and adult AChRs. However, desensitization time constants tended to be slower in the muscle‐type cell line compared to non‐muscle cells. Differences in post‐translational AChR modification or the interaction of AChRs with other proteins (Burden *et al*. 2018) differently expressed in the two cell lines could have contributed to the observed variations in TE671/CN21 and HEK293 cells. Consequently, TE671/CN21 cells were used in experiments with respect to the effect of rapsyn on AChR function to ensure a more physiological intracellular environment for AChRs. C2C12 myotubes are capable of producing well‐formed AChR clusters upon agrin stimulation and thus may qualify as an even better model for experiments on many aspects of muscle function, but their utilization for measurements of recovery from desensitization by whole‐cell patch clamping is associated with various methodological problems as a result of the large cell size and large current amplitudes (Traynelis, 1998; Fu *et al*. 2011). Moreover, the study of mutant AChRs or proteins involved in AChR clustering is limited by the endogenous expression of the wild‐type form of those proteins in C2C12 myotubes. In our experiments on chimeric AChRs and the δ‐R375H mutation, we thus used HEK293 cells that do not express wild‐type AChRs, the presence of which would have made the findings uninterpretable. As a limitation, there are obvious differences in AChR clustering between the three cell lines utilized in our experiments, which might compromise the comparability of some of the results. The most obvious explanation for these variations is the lack of the molecular machinery in TE671/CN21 and HEK293 cells required for full AChR clustering. However, there can be little doubt from the evidence obtained here that the co‐transfection with rapsyn is at least sufficient to form rudimentary AChR aggregates, which differ functionally from AChRs without rapsyn. Evidence also derives from experiments on myasthenia gravis, in which some previously AChR antibody negative sera were shown to contain IgG antibodies that bound AChRs expressed on a cell surface only when co‐transfected with rapsyn (Leite *et al*. 2008; Rodriguez Cruz *et al*. 2015); this is now used as a clinical assay in several laboratories.

To our knowledge, no previous studies have investigated the consequences of clustering on AChR function, which is an essential characteristic of the normal NMJ. Intriguingly, rapsyn co‐transfection into both muscle‐type and non‐muscle cell lines, which induced clearly visible aggregates on the surface of the cells, facilitated recovery kinetics of both fetal and adult AChRs. More surprising, however, was that rapsyn increased recovery rates even in AChRs that were unable to cluster in C2C12 myotubes because of a mutation in the delta‐subunit (δ‐R375H), suggesting a direct effect of rapsyn on AChR function independent of receptor clustering. Alternatively, AChRs harbouring the δ‐R375H mutation could have aggregated into mini‐clusters of reduced size not visible by microscopy but sufficient to facilitate AChR recovery from desensitization.

The small AChR aggregates in HEK293 cells were clearly different compared to the large AChR clusters in C2C12 myotubes and might thus represent different structural modifications of the AChR surface distribution. One potential explanation for the variation of AChR aggregation and clustering between both cell lines is the lack of the molecular machinery in HEK293 cells that is required for full AChR clustering. It is also important to appreciate that the effect of rapsyn on HEK293 or TE671/CN21 cells cannot be considered identical to the effects in C2C12 myotubes or even at the NMJ. The relatively small size of these cells ranging between 10 and 20 µm would probably not accommodate AChR clusters of around 10 µm typically seen in C2C12 myotubes and certainly not a full‐sized mature NMJ of up to 30 µm in human muscle. Because the interactions between AChRs and rapsyn do not form the typical AChR clusters described in C2C12 myotubes, we refer to these as AChR aggregates rather than clusters. However, our finding of allosteric modulation of AChR function by its interaction with rapsyn is interesting and may also be relevant for other proteins expressed at the NMJ, which were shown to directly interact with the AChR. These include Src‐family kinases (Fuhrer and Hall, 1996), the tumour suppressor protein adenomatous polyposis coli (Wang et al. 2003) and caveolin‐3 (Hezel et al. 2010).

The physiological significance of desensitization and recovery from desensitization at the NMJ is disputed (Giniatullin *et al*. 2005). Desensitization is a slow process compared to the lifetime of ACh in the synaptic cleft that ranges between 200 and 500 µs (Smart & McCammon, 1998) as a result of hydrolysis by the enzyme acetylcholine esterase (AChE). Therefore, desensitization might not have a significant impact on shaping endplate currents in muscle. However, desensitization might become relevant at highly active synapses. Motor neuron firing rates are extremely low, around 0.5 Hz, in mice at birth when fibres are still multiply innervated but, from the second week, there is an increase of motor neuron firing frequencies to levels usually found in adult mice (Vrbova *et al*. 1985; Personius & Balice‐Gordon, 2001). These high motor neuron firing rates, however, can overcome the catalytic AChE capacity and increase the lifetime of ACh in the synaptic cleft sufficiently to desensitize AChRs (Magleby & Pallotta, 1981; Ruzzier & Scuka, 1986; Giniatullin *et al*. 1993; Giniatullin *et al*. 1997), leading to a progressive reduction in endplate currents. In this context, the developmental switch to the ε‐subunit with faster recovery kinetics, which occurs around the same time as the change in motor neuron firing rates, could provide an adaptation to increasing motor neuron firing rates after birth, facilitating the robust and reproducible high‐frequent neuromuscular transmission in the adult. The effects of rapsyn on recovery times, as well as the switch from fetal to adult AChRs, would be important to help maintain a sufficient number of activatable receptors between stimuli at high frequent firing rates.

These results have potential clinical relevance. Rapsyn‐induced clustering of AChRs is the result of a highly regulated pathway initiated by nerve‐released agrin, interacting with LRP4 and stimulating MuSK and DOK7 phosphorylation, which then leads eventually to rapsyn and AChR clusters. There are a number of genetic disorders of the neuromuscular junction (CMS), many of which are caused by mutations in the AChR, rapsyn or DOK7 but associated variably with extraocular muscle weakness. Extraocular muscles have exceptionally high motor neuron firing frequencies in the range of several hundred hertz (Yu Wai Man *et al*. 2005), which therefore require fast AChR recovery rates for efficient neuromuscular transmission. In patients with null mutations of the CHRNE gene (coding for the ε‐subunit) (Ealing *et al*. 2002; Engel *et al*. 2015), neuromuscular transmission has to depend on the fetal AChR, and one major symptomatic feature of these patients is ophthalmoplegia. Rapsyn‐induced clustering of the fetal form and its effect on recovery from desensitization could be crucial with respect to allowing a number of these patients to have some, albeit impaired, extraocular muscle function. Further investigation of the role of rapsyn could help in understanding better the pathophysiology of these very heterogeneous and interesting synaptic disorders.

In conclusion, our findings provide important justifications for the significance of the developmental switch from fetal to adult AChRs around the time of birth, and they are also relevant to patients with CMS where there is complete loss of adult AChRs and where low levels of fetal AChR expression are sustained in mature muscle.

## Additional information

### Competing interests

The authors declare that they have no competing interests.

### Author contributions

All experiments were carried out in laboratories of the MRC Weatherall Institute of Molecular Medicine, University of Oxford, John Radcliffe Hospital, Headington, Oxford, UK. HC, AV, DB and RW designed the research. HC, WL, JC, JC, AV and SM performed the research. HC analysed data. HC wrote the paper. All authors have read and approved the final version of the manuscript submitted for publication. All authors agree to be accountable for all aspects of the work in ensuring that questions related to the accuracy or integrity of any part of the work are appropriately investigated and resolved. All persons designated as authors qualify for authorship, and all those who qualify for authorship are listed.

### Funding

We are grateful for financial support offered by the following funding agencies: HC was funded by an Erwin Schrödinger Fellowship (J3589) by the Austrian Science Fund (FWF) and by a grant from the Nuffield Department of Clinical Neurosciences, University of Oxford. DB holds a Medical Research Council Program Grant (MR/M006824/1), which funds RW.
